# Supplementary material for: Effects of Biological Nitrogen Metabolism on Glufosinate-Susceptible and -Resistant Goosegrass (Eleusine indica L.)
Source: Int J Mol Sci. 2023 Sep 7;24(18):13791. doi: 10.3390/ijms241813791 (PMC10531271; doi:10.3390/ijms241813791)
Supplement: Supplementary file 1 [file ijms-24-13791-s001.zip › ijms-2531222-supplementary.pdf]

Article

Effects of biological nitrogen metabolism on glufosinate-susceptible and resistant goosegrass (*Eleusine indica* L.)

Figure S1. General analysis on characterizations of glufosinate-resistance goosegrass by proteomic sequencing. (a)-(h) Enrichment scatter plots of KEGG pathways in the eight comparative groups of the different goosegrass biotypes (NX, CS, and AUS) under glufosinate stress or under H_2O as the controls.

(a)

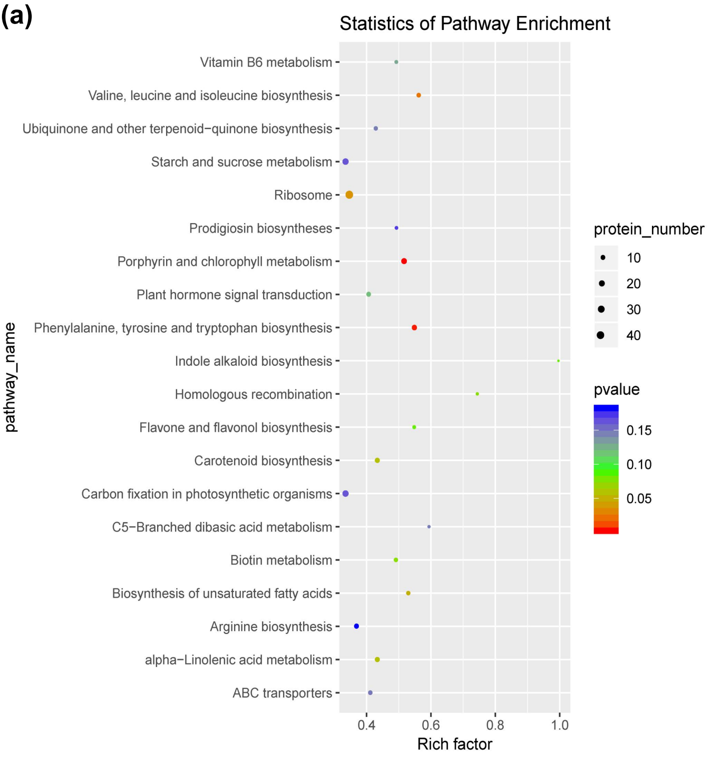

CK-AUS vs. CK-CS

(c)

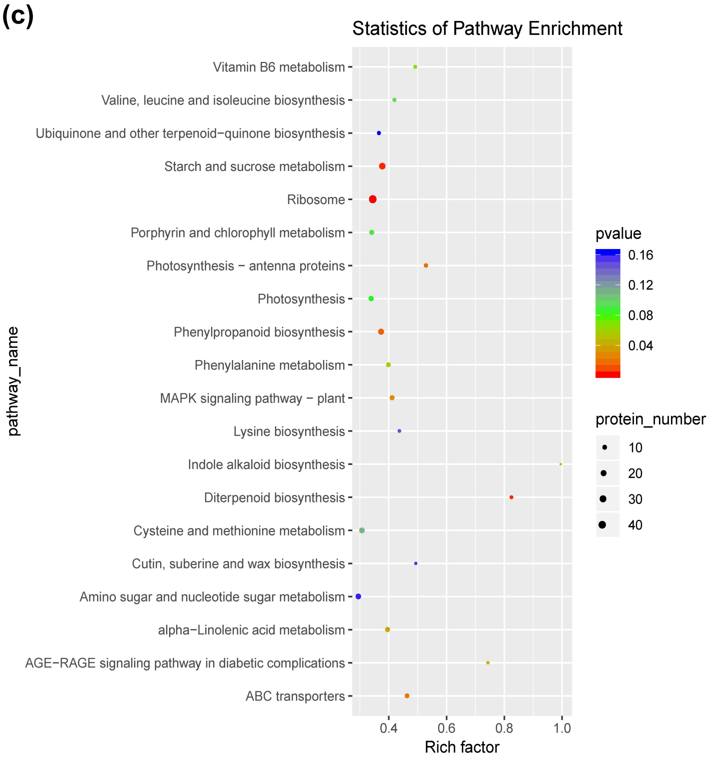

T-AUS vs. T-CS

(b)

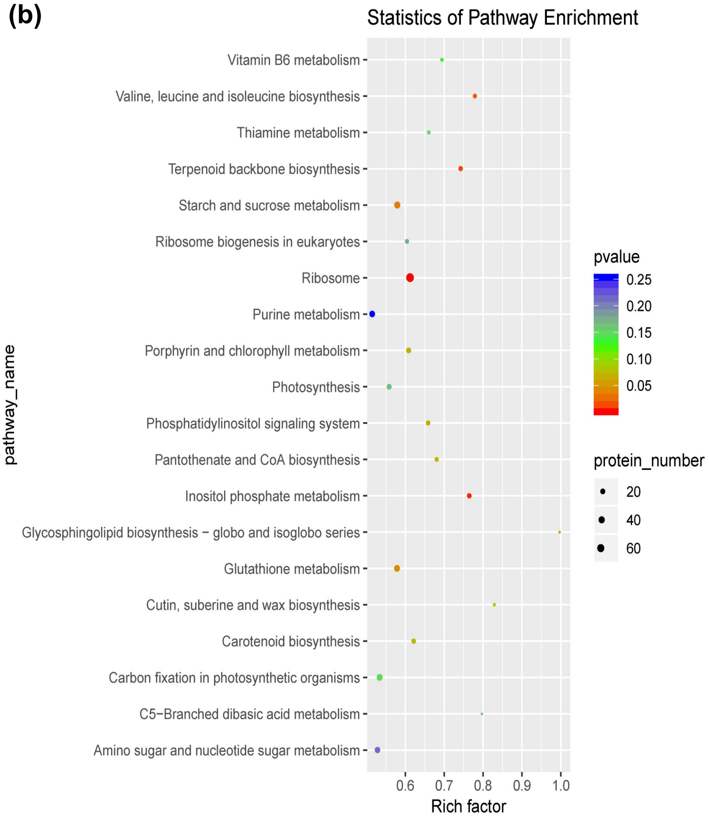

CK-AUS vs. CK-NX

(d)

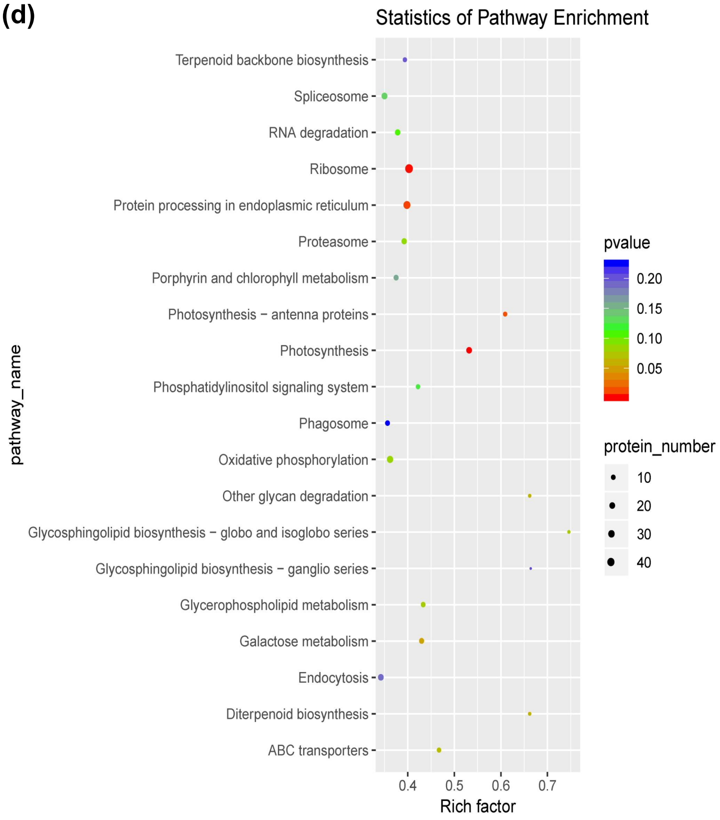

CK-CS vs. CK-NX

(e)

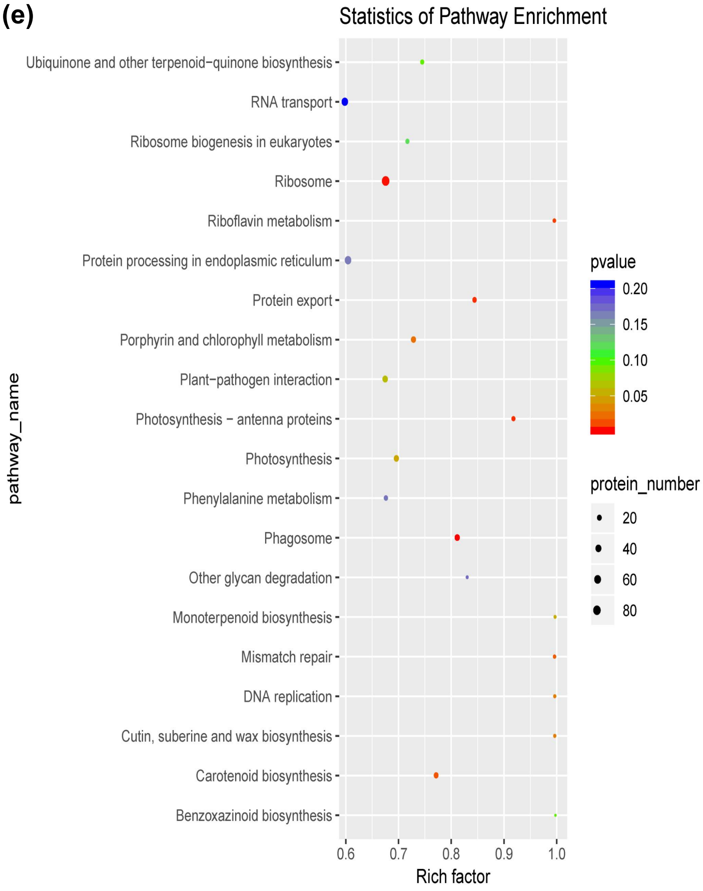

T-AUS vs._CK-AUS

(g)

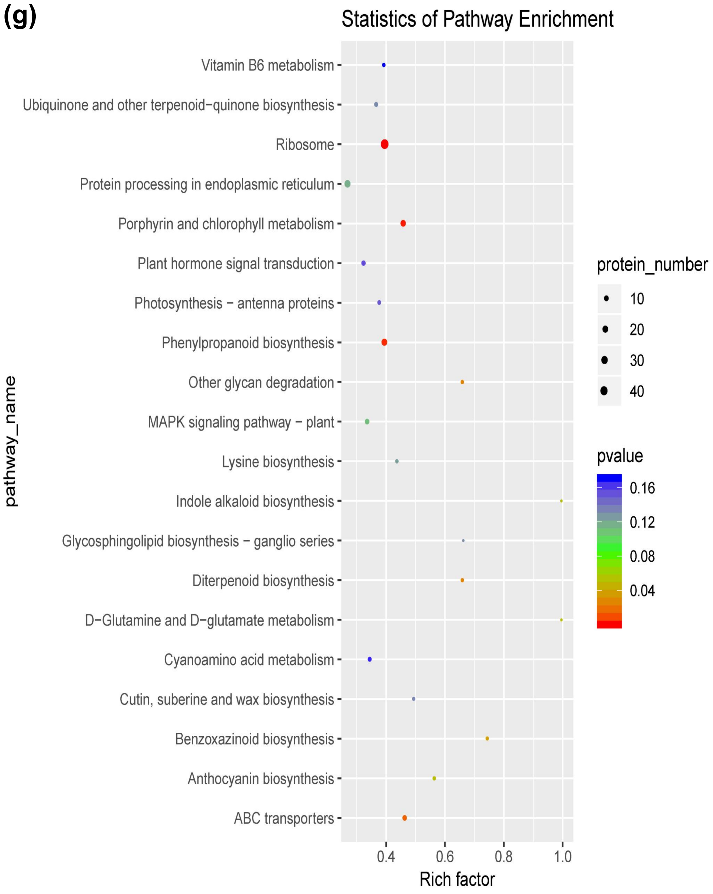

T-AUS VS. T-NX

(f)

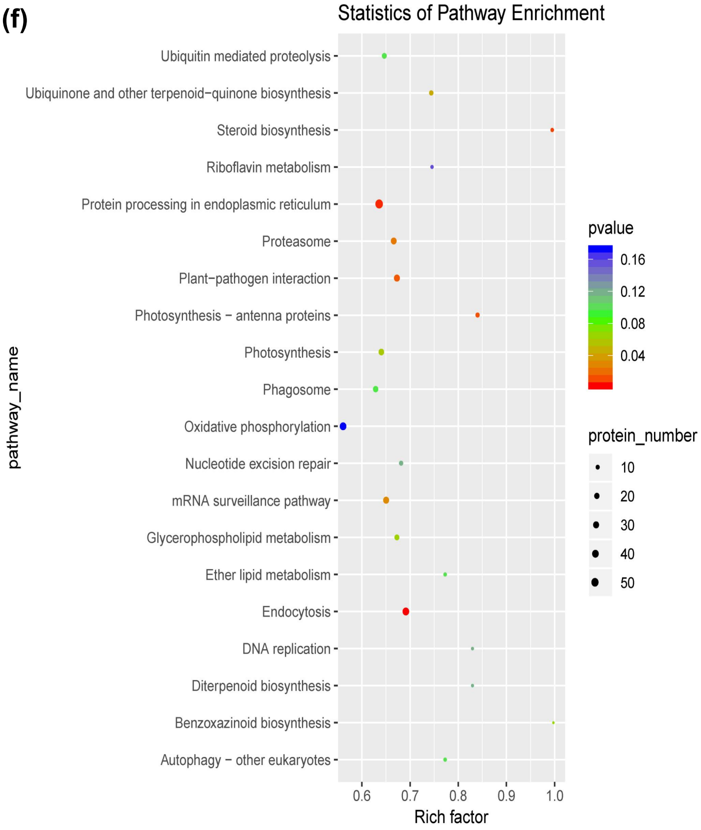

T-CS vs. CK-CS

(h)

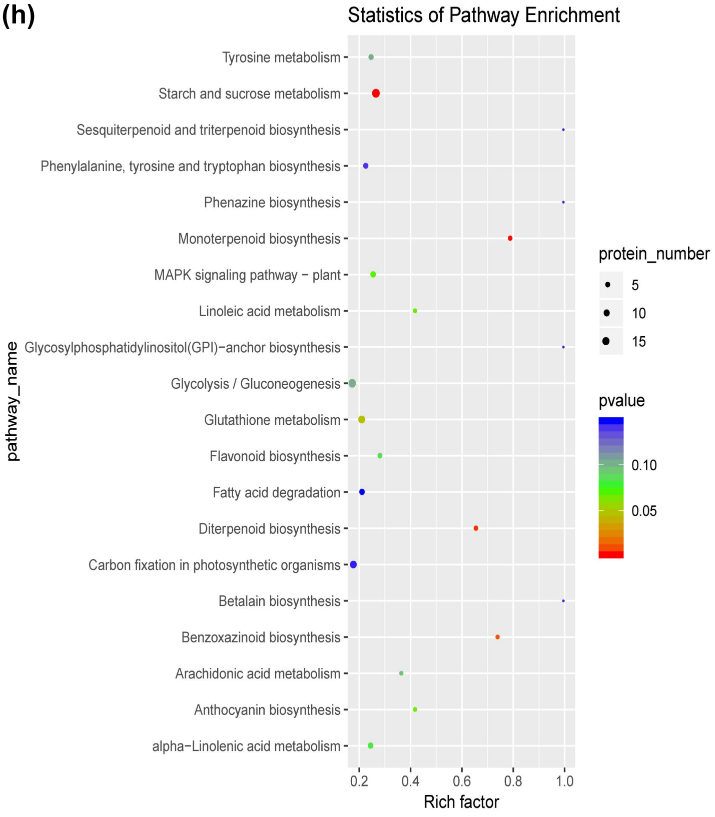

T-CS vs. T-NX

Figure S2. Differential expressions of genes and proteins in KEGG pathways of nitrogen metabolism. (a) and (b) Transcriptome and proteome analyses in the comparative group T-NX vs. CK-NX. (c) and (d) Transcriptome analyses in the comparative group T-AUS vs. CK-AUS and T-CS vs. CK-CS, respectively.

(a)

Transcriptome

T-NX vs. CK-NX

(b)

Proteome

T-NX vs. CK-NX

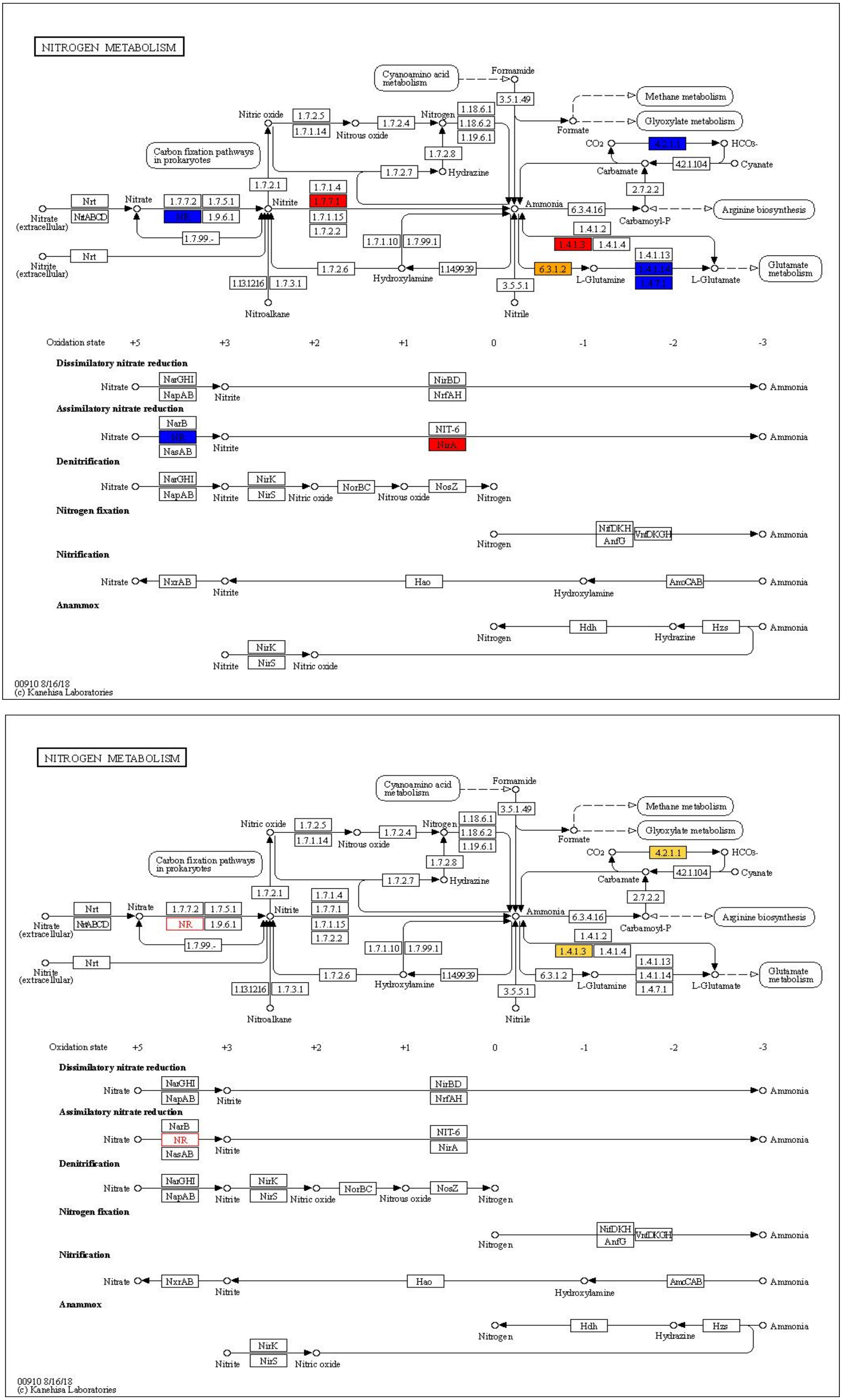

(c)

Transcriptome

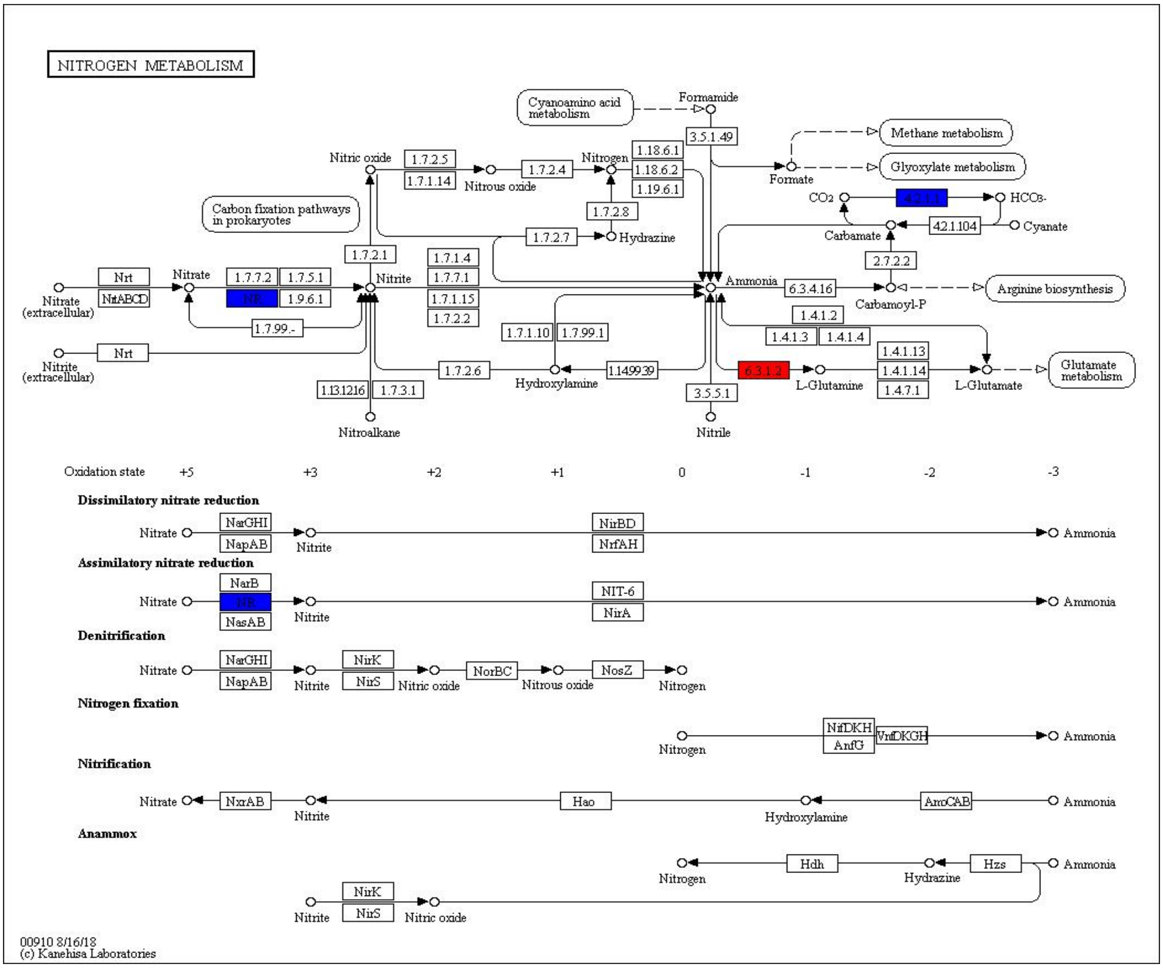

T-AUS vs. CK-AUS

(d)

Transcriptome

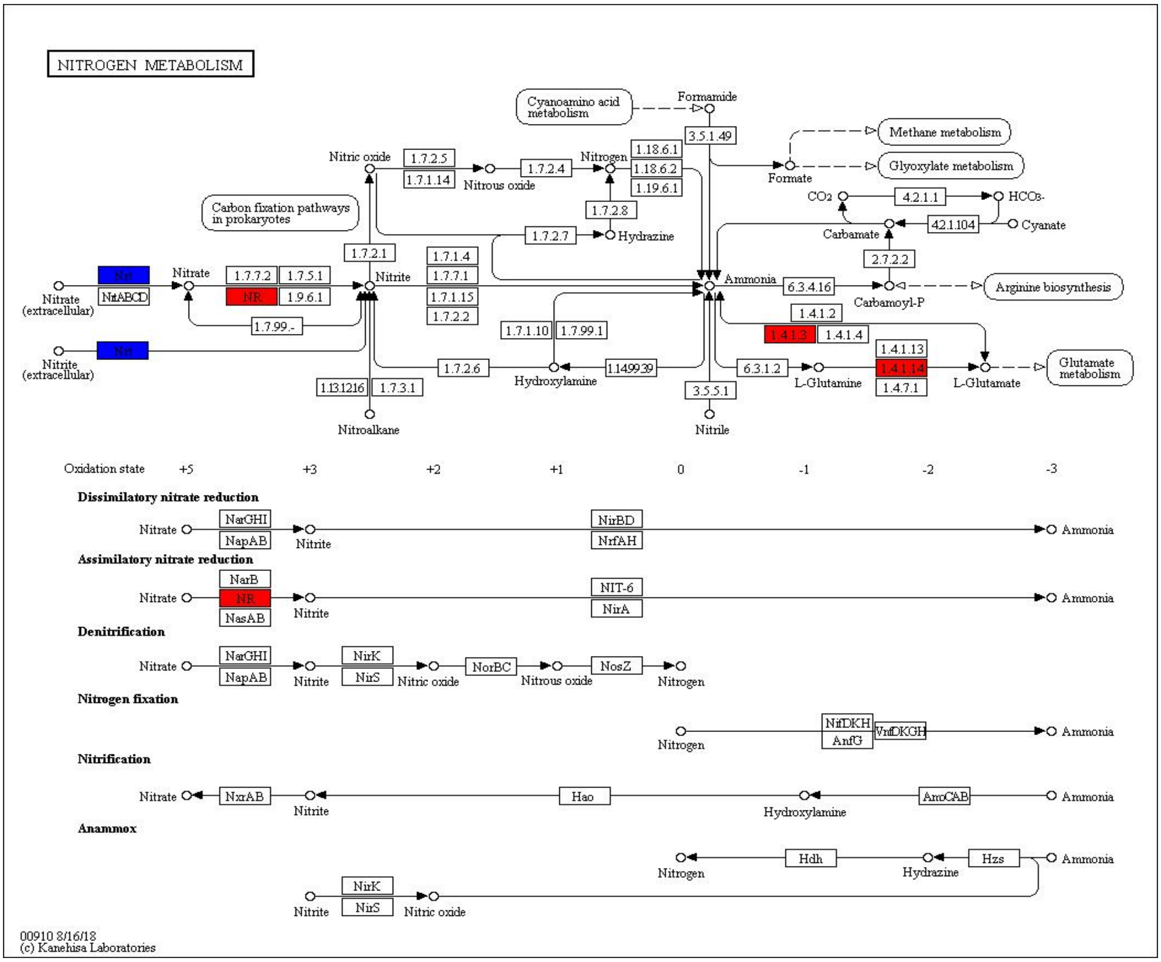

T-CS vs. CK-CS

Figure S3. The CDS sequences of *GLN1-1*(a), *GDH2*(b), *GLSF*(c), *NIA1*(d), *Os01g0357100*(e) and *CA1*(f).

(a) TRINITY_DN25503_c1_g2 Trans_ID=TRINITY_DN25503_c1_g2_i2 1414bp (***GLN1-1***)

CATCTCCTTCCTCCTCCTCCTCCATCCCCAACCCGTCTGCTTTGTCTGTGTACCACCCCAGCCGCCGGCCATGGCCTGCCTCACCGACCTC
ATCAACCTCGACCTCTCCGACTCCACCGAGAAGATCATCGCCGAGTACATATGGATCGGTGGATCTGGCATGGACCTCAGGAGCAAG
GCGAGGACCCTCCCCGGCCCGGTGAGCGATCCCAGCAAGCTGCCCAAGTGGAACTACGACGGCTCCGGCACCGGCCAAGCTCCCGG
CGAGGACAGTGAGGTCATCCTATACCCACAGGCCATCTTCAAGGACCCATTCAGGAAGGGCAACAACATCCTTGTCATGTGCGATTGC
TACACCCCAGCTGGCGAGCCAATTCCCACCAACAAGAGGGCCAACGCTGCCAAGATCTTCAGCAACCCTGAGGTTTCTGCTGAGGAG
CCCTGGTACGGTATTGAACAGGAATACACCCTCCTGCAGAAGGACACCAACTGGCCTCTTGGGTGGCCTCTTGGTGGCTTCCCTGGTC
CACAGGGTCCCTACTACTGCGGTGTTGGCGCTGACAAGTCATTCGGGCGTGACATTGTTGACTCCCACTACAAGGCTTGCCTGTATGC
CGGAATCAACATCAGTGGCATCAATGGTGAAGTCATGCCAGGACAGTGGGAGTTCCAAGTCGGCCCTGCTGTTGGAATTTCTGCTGG
TGACCAGGTGTGGGTTGCTCGCTACATTCTTGAGAGGATCACTGAGATCGCCGGTGTCGTGGTGACATTCGACCCCAAGCCTATCCCT
GGTGACTGGAACGGTGCCGGTGCTCACTCAAACTACAGCACCAAGTCCATGAGGAACGACGGCGGCTACGAGGTGATCAAGTCCGC
GATCGAGAAGCTCAAGCTGAGGCACCGGGAGCACATCGCCGCCTACGGTGAGGGCAACGAGCGCCGGCTCACCGGCAAGCACGAG
ACCGCTGACATCAACACCTTCAGCTGGGGCGTGGCAAACCGTGGCGCGTCTGTGCGTGTGGGTCGCGAGACGGAGCAGAACGGCAA
GGGCTACTTCGAAGACCGCCGGCCGGCGTCCAACATGGACCCCTACGTGGTGACCTCCATGATCGCCGAGACCACCATCATCTGGAA
GCCTTGATCGCGTCCCACCCTCACCAGGATTTGGCGATTGCATTGTGCCCCGGGGGAAACAATTCGTCTTCTGATTCACGCAAAGCATC
TCATTGTCTCCTGTTCCTGTCCCATTTGGTTACACTGCTACTATTACTACAGCATTTAGTTAGGTCTGGGTATGTTCATTTCACCTCGGGG
TGAGTGATGATCATGGTGGCAAGTGGTGTGAAGGACAAAGACATTGTTGCTTCCTTGCCGTCCGTTTGGGTGGTCCTGTGTAATCCAA
TAATGGCCGTAC

(b) TRINITY_DN23079_c2_g5 Trans_ID=TRINITY_DN23079_c2_g5_i1 2083bp (***GDH2***)

CGATGATGAATCAGCGCGAGACTCCCTCTCCCAGAAATTTCCTACCGCGCTCGATCTCTCTTCCGTATCTGGCATTAGTGCGCGCATTA
ACGGATTCCCGTTTTAATTAATCTCTCCCGCCCGCCTGGAAGACGTGCAGCGGGTTCCAATCGAGCCCGAGTCGCGCTCCAGCATTTCC
CTACTTCCTATAGCGAGCAGTTCTACCCGGCTTCCAAATTCCTTCCCGCTGCAACAAGAGTTCCAGATCGAGAAGCCAGCGGAGCCAC
CTCGAGTCCTTGTTCTTCTTCTACCCAGCTCGGCGATCTTATCCTCCTTCCACCTCTGGCTGGATCCCTATTAATATCACGCGCCTCAGCT
TGCCCCCAATCCATCGCTCTCGCCGTGGCTCGTTTCCTCGATTCACGGAGAAGAAAACGCCGCCTTTTCCCTTTGCCGTTTTTGGCTCGC
CCCGATCTCGCTCGCGTCGCGTGCGCTTGTCCCTGCCCCCGTGCGCTGCGTGTGCTGTTCTGTCTGAAAGGCGGGTCCTTTCTTCCGCC
GCCGCCCGCTCGTGATCCTTGTCGGAGCTGAGAAAGGTGCGACCATGAACGCGCTCGCCGCCACCAGCCGCAACTTCCGGCAGGCCG
CGAGGCTGCTCGGCCTCGACTCCAAGCTCGAGAAGAGCCTCCTCATCCCGTTCCGTGAGATCAAGGTGGAATGCACCATCCCCAAAGA
CGACGGCACCTTGGCGTCGTTCATCGGATTCCGCGTGCAGCATGACAATGCCCGCGGGCCGATGAAAGGCGGTATCCGCTATCACCC
TGAGGTTGAACCTGATGAAGTAAACGCACTTGCGCAACTGATGACATGGAAGACCGCAGTTGCAGCAGTACCATATGGTGGAGCAAA
GGGAGGGATTGGATGCTCTCCTAGTGAACTAAGTAGAAGTGAGTTGGAGCGGTTGACACGTGTGTTCACGCAGAAAATTCATGATCT
TATCGGTACCCATACAGATGTACCAGCTCCAGACATGGGGACAAATTCACAAACCATGGCATGGATTTTAGATGAGTATTCAAAATTT
CATGGCCATTCCCCAGCAGTTGTCACAGGGAAGCCAATAGATCTTGGCGGATCATTAGGCAGGGATGCTGCAACTGGACGAGGTGTG
ATGTATGCTACAGAGGCTCTGCTGGCTGAACATGGAAAGTCCATTGCTGGATCAACTTTTGTCATTCAGGGGTTTGGTAATGTTGGTTC
ATGGGCTGCACAACTTATTCATGAGAAAGGTGGTAAAATAATTGCACTTGGAGATGTCACTGGCTCAATCAGAAGCAAGAGTGGCAT
AGACATACCTGCACTGATGAAGCACAGGGATGAGGGTGGTGCTTTGAAAGATTTCCATGGTGCGGAAGTTATGGATTCTTCTGAGTT
GCTAGTGCATGAATGTGATGTTCTTATGCCGTGCGCGTTAGGTGGAGTTCTTAACAGGGAAAATGCGCCACATGTGAAGGCCAAGTTT
ATAGTTGAAGCTGCTAACCATCCAACTGATCCAGAGGCAGATGAGATTTTTGCCAAGAATGGAGTGACAGTATTGCCAGATATCTATG
CCAATTCTGGTGGTGTGATTGTTAGCTACTTTGAATGGGTTCAGAACATTCAAGGCTTCATGTGGGAAGAAGAGAAGGTGAACATGG
AACTCCACAAGTACATGAACAACGCTTTTCATCACATCAAGGCCATGTGCAAGTCTCAAGAGTGCAACCTTAGAATGGGGGCATTCTC
CTTAGGAGTTAATAGGGTTGCCCGTGCCACCTTGTTGAGGGGCTGGGAAGCATGAGATCGTTCCATACATATAGCAGTTTCGCCGTTC
AATAATTTTGCATTCTACATTGTTTGTGGCCCATGTCTGATCTGGTAAAATTATCAGAGGTGTTTTGCATGGATTGAAACCAGTTGAAAT
AATATGCTGGATACATGGCGAATGTGTTGTGTTTGTTCTTATTTCCTCTGATTTCCTGGGTTGGCAATGTTGGTATTCAGTTTTGGATGT
CGATAATTCGCATTATGGGGAACCCCGTAGTTTGTTCTATGCTTCAGAGATGCC

(c) TRINITY_DN23865_c0_g14 Trans_ID=TRINITY_DN23865_c0_g14_i1 5055bp (***GLSF***)

GCCTGTGCAGGAGGCTACAGATTTGAATGATATCTTATCTGAACGTGGAGCCTGCGGTGTTGGTTTTGTTGCAAACTTGAGGAATGT
GCCTTCATTCAACATTGTTCGTGATGCTCTTATGGCTCTTGGGTGCATGGAGCACCGTGGTGGCTGTGGAGCAGACAATGACTCTGG
TGATGGGTCAGGACTGATGAGCGGTATTCCATGGACTTGTTTGATGACTGGGCCAACAAGCAAGGACTTGCTCCCTTTGACAGAAG
AAATACAGGTGTAGGCATGGTTTTCCTTCCAAAAGATGAGAAGTCCATGGCAGAAGCCAAAGCCGTAACTGAAAAGGTTTTTAAGG
ATGAAGGCCTTGAGGTTCTTGGATGGAGAGGTGTTCCTTTTAATGTAGCAGTGGTAGGTCGCAATGCAAAAGAGACCATGCCTAATA
TTGAACAAGTATTTGTAAAAGTTGCAAAAGAAGATAATGCTGATGACATAGAGAGGGAATTGTACATCTCCCGGAAGCTGATAGAG
CGGGCTGCAAAATCTGAAAGTTGGTCAGATGAACTTTATTTTTGCTCTTTGTCAAGCAGAACCATTGTCTACAAGGGAATGCTCCGAT
CTGAGGTTCTTGGGCAATTTTATTTGGACCTTCAGAATGAACTGTACAAATCTCCTTTTGCCATATATCATCGAAGATACAGTACAAAT
ACAAATCCTAGATGGCCTCTTGCTCAACCTATGAGGTTGCTTGGACACAATGGAGAAATTAACACAATACAGGGGAACTTGAACTGG
ATGCGATCGAGGGAAGCCACACTACAATCTCCAGTATGGCGGGGCCGTGAGCCTGAAATACGTCCGTTTGGTGACCCTAAAGCATCT
GATTCAGCAAATCTTGACAGCACCGCTGAATTACTGCTAAGGAGTGGTAGAAGTCCCGCTGAAGCTCTTATGATTCTCGTTCCTGAGG
CATATAAGAACCATCCTACATTATCAATAAAATATCCTGAGGCAGTTGACTTTTATGACTACTACAAAGGCCAAATGGAGGCTTGGGA
TGGACCTGCTTTACTATTATTCAGTGATGGAAAGACAGTAGGAGCAACTCTTGATAGAAATGGGCTCCGTCCAGCACGCTATTGGAG
AACATCAGATGATTTTGTTTATGTTGCATCTGAGGTTGGTGTTATACCAATGGATGAGTCAAAGGTAGTAATGAAAGGAAGATTAGG
ACCTGGAATGATGATAACTGCTGACCTAGAGACTGGACAGGTGCTTGAAAACACAGAGGTGAAGAAAAATGTGGCTTCTGCATACC
CCTATGGAACCTGGTTGCAGGAACGTACACGCTATATAAAACCTGTCAACTTCCTCTCTTCAACCATCATGGACAATGAGACGGTTTT
GAGACATCAACAGGCATTTGGCTATTCTAGTGAAGATGTGCAATTGGTAATTGAATCAATGGCTTCACAAGGAAAGGAACCAACATT
TTGCATGGGCGATGACATTCCATTAGCCGTGTTGTCACAAAAGCCGCACCTGTTATATGATTATTTCAAGCAGCGGTTTGCACAGGTA
ACAAATCCTGCCATTGATCCGCTCAGAGAAGGTTTAGTTATGTCTCTCGAAGTTAATATCGGTAAAAGAGGCAACATCTTGGAGGTT
GGGCCTGAAAATGCTGATCAGGTTGCTCTATCAAGTCCTGTGCTGAATGAAGGTGAACTAGAATCGCTATTGAAGGACCCGACACTG
AAGCCCAAAGTACTCTCAACGTACTTTGATATTCGTAAAGGTCTAGATGGTTCTCTTGACAAGACAATTAAGGCTCTTTGTGAAGAAG
CTGATGCTGCTGTGCGGAGTGGTTCTCAACTTCTTGTGCTTTCTGATCGTTCTGAAGAACCTGAACCAACTCGGCCTGCCATACCAAT
ACTTCTAGCTGTTGGTGCCATCCACCAGCATTTGATTCAGAATGGCCTTAGAATGTCGGCCTCTATTGTTGCTGATACTGCTCAGTGTT
TCAGCACCCATCACTTCGCCTGTTTGATTGGATATGGAGCCAGTGCTGTGTGTCCTTATCTGGCACTGGAGACATGCCGGCAATGGA
GGCTGAGTAACAAAACAGTTAATTTGATGCGAAATGGGAAAATGCCCACTGTGACCATCGAGCAGGCTCAAAGAAACTTTATCAAG
GCAGTGAAATCTGGTCTGCTCAAGATACTTTCAAAAATGGGCATCTCACTGCTCTCAAGTTACTGTGGAGCTCAGATCTTTGAAATTT
ATGGCCTTGGTCAAGAAGTTGTTGATCTTGCCTTCTGTGGAAGTGTATCCAAAATTGGAGGGCTCACACTGGACGAGCTGGGGAGA
GAAACTTTGTCATTCTGGGTGAAGGCATTCTCAGAAGATACTGCAAAGAGGCTAGAGAACTTTGGATTCATTCAATCCAGACCTGGA
GGTGAATACCACGCAAATAATCCTGAGATGTCAAAGCTGCTGCACAAAGCAATTCGCGAAAAAAGAGATAACGCATATACCGTCTAC
CAACAACACCTTGCAAGTCGTCCTGTCAATGTTCTTCGAGATCTTCTTGAATTGAAGAGTGATCGTGCACCTATTCCTATTGGCAAAGT
TGAGCCTGCAACCTCCATTGTTGAGCGTTTTTGTACAGGTGGAATGTCCCTAGGAGCCATTTCAAGAGAAACACATGAAGCTATTGCA
ATCGCAATGAATAGAATAGGCGGTAAATCTAACTCGGGAGAAGGTGGTGAGGACCCGATCCGTTGGAATCCCCTCACAGATGTTGT
AGATGGGTATTCTCCAACACTTCCTCATCTCAAAGGTCTTCAGAATGGTGACACTGCCACAAGCGCCATTAAGCAGGTTGCATCTGGG
CGTTTTGGAGTTACACCAACCTTTTTAGTAAATGCTCAACAAATTGAGATAAAGATTGCACAAGGTGCAAAGCCTGGTGAAGGGGGT
CAACTTCCTGGGAAAAAAGTCAGTGCATATATTGCTAGGCTGAGGAACTCCAAACCTGGAGTTCCTCTCATATCCCCACCCCCACACC
ATGACATCTATTCTATTGAGGATCTTGCACAGTTAATTTATGACCTTCATCAGATCAATCCTAAAGCCAAGGTCTCCGTAAAGCTTGTA
GCTGAAGCTGGGATTGGAACTGTAGCTTCTGGAGTTTCCAAGGGAAATGCAGATATTATTCAGATATCAGGCCATGATGGTGGTACA
GGAGCTAGCCCAATCAGCTCAATCAAGCATGCTGGGGGTCCTTGGGAACTTGGTCTTACAGAAACGAATCAGACACTTATACAAAAT
GGATTGAGAGAGAGGGTGGTACTTAGGGTGGATGGTGGATTCAGGAGTGGCCAGGATGTTCTTATGGCTGCTGCCATGGGCGCTG
ACGAATATGGTTTTGGTTCTGTAGCTATGATAGCCACAGGATGTGTCATGGCACGCATTTGCCACACAAATAATTGCCCAGTTGGAGT
TGCTAGTCAGAGAGAAGAGCTTCGTGCCAGGTTTCCTGGTGTTCCCGGTGATCTTGTTAACTATTTCCTCTTTGTTGCAGAGGAGGTA
CGGGCTGCATTAGCCCAACTGGGTTTCGAGAAGCTGGATGATATAATTGGGCGTACAGATTTGCTTAAGCCGAAGCATGTCTCTTTG
GTGAAAACACAGCACATTAACCTTGGATACCTCTTAGAAAATGCTGGATTACCCAAATGGAGCAGCTCACAGATTAGGAGCCAGGAC
GTCCACACTAATGGCCCTGTGCTTGATGAGACAATCCTTGCGGATCCTGAGATAGCTGATGCTATAGAGAATGAGAAAGAGGTTTCA
AAGACATTTCAAATTCATAATGTTGATAGAGCTGTGTGTGGCCGAGTAGCGGGTGTGATTGCCAAGAAGTATGGAGACAGAGGTTT
TGCAGGGCAGCTTAACGTCACGTTCACTGGAAGTGCGGGACAGTCCTTTGGTTGTTTCTTGACTCCAGGAATGAATGTTCGGCTAGT
TGGAGAGGCCAATGATTATGTCGGAAAGGGTATGGCTGGTGGAGAACTAGTAGTAGTTCCTGTAGAAAATACAGGATTTGTTCCTG
AGGATGCTACTATAGTTGGAAACACTTGTCTGTATGGAGCCACAGGTGGTCAAGTATTTGTGAGAGGCAAGGCAGGAGAAAGATTT
GCAGTTAGAAACTCTCTAGGTCAGGCAGTGGTTGAGGGCACTGGAGATCACTGCTGCGAGTACATGACTGGTGGCTGTGTAGTTGT
ACTTGGCAAAGTGGGAAGGAATGTTGCTGCTGGAATGACTGGTGGCCTTGCTTACATTCTAGATGAGGATGATACACTCACACCAAA
GGTCAACAAGGAAATTGTCAAGATGCAGAGAGTGAATGCTCCAGCTGGGCAGATGCAGCTGAAGAGTTTGATTGAGGCTTACGTTG
AAAAAACGGGCAGTGAGAAAGGTGCCACGATTTTGAGGGAATGGGAGGCATATTTGCCACTCTTCTGGCAACTGGTACCGCCCAGC
GAAGAAGACTCACCTGAGGCCTGTGCCGAGTTTGAGAGAGTACTCGCTAAGCAAGCAACCACCCAGCTATCTGCGAAGTGATCAAA
TCAGGCAACAGGAAGGTGTGCTCTTTCTGACACTCACATAGTGATTTCCGCTGACCGCTTCAAGAAAAAAGTAACCGGCATACTTCTG
AAGCACCGAAGTGCCCGATGGCTGCAATTTTTTTGTGGAGGCTCTGCTCAAGTCATCTTTTTGGAGGCTCTGAGCTCGCAGAACCAT
CGTGGATTCATCTCTGACGCCCTTGCATCCTAGGTAACCCCTGTAAATTGGTTAACTGGCGCCAGGTTGCAAGGGCGCTCTTTGGAAC
TGTTGCATTCTTGTACATGATTCTTAGCTCTTGTCCCGTGTAATTATGTTTTGGTGAGCTGTTAAATTTATACGGATCCAGTGGAGGCA
TGCAAGCGGCATGAGCATGTGGTACTTGTACATAATAAGTTGTACGGTTCAGCAGGGTATCTCAGTCTGCTTTCATGAATGAACG

(d) TRINITY_DN24663_c0_g1 Trans_ID=TRINITY_DN24663_c0_g1_i1 3174bp (***NIA1***)

CAGCTAGCGCCTCTTCACAAGCATCTCACAGAAAGCAGAGAGATCGGACCTCTAGCACTACACACGCCCAGGTCTCCCTGACATGGCCG
CTTCTGTCGAGCCACGGCAGTTCGGCCGCCTCGAGCCTGGTTCCGGGCAGGTCCGCGTGGCCACGAACGGCGCCAAGGCGTACCCTCC
CCCGGCGAGCCACATCCCCCGCCGCTCCGACTCGCCCGTGCGCGGCTGCGGCTTCCCTCCGCTCGTTTCCCCGCCGCGGAAATCCGATG
ACGCTTCGGACGACGACGACGACGAGCCGGAGGACTGGCGCGAGCTGTACGGCTCGCACTCCCAGCTGGAGGTTGAGCCGGCGGTG
CACGACCCGCGCGACGAGGGCACTGCCGACGCGTGGATCGAGCGCAACCCGTCGCTCATCCGGCTGACGGGGAAGCACCCGCTCAAC
TGCGAGCCGCCGCTGGCGCGGCTGATGCACCACGGCTTCATCACCCCGGCGCCGCTGCACTACGTGCGCAACCACGGCGTGGTCCCCA
GGGGCGAGTGGGCGACGTGGGCCGTGGAGGTGACGGGCCTCGTGAAGCGGCCCGCGCGGCTGAGCATGGACGAGCTGGTCCGCGA
CTTCCCCGCCGTGGAGGTGCCCGTGACGCTGGCGTGCGCGGGCAACCGGCGCAAGGAGCAGAACATGGTGCAGCAGACGGTGGGGT
TCAACTGGGGCGCCGCGGGCGTGTCCACCTCCGTGTGGCGCGGCGCCAGGCTCCGCGACGTGCTGCGCCGATGCGGCATCATGCCGC
GCAAGGGAGGCGCGCTCAACGTGTGCTTCGAGGGCGCAGAGGACCTGCCCGGCGGCGGAGGGTCCAAGTACGGGACCAGCGTCACC
AGGGAGTGGGCGCTCGACCCGTCCAGGGACATCATGCTCGCCTACATGCAGAACGGGGAGCCGCTGCTGCCCGACCACGGGTTCCCG
GTTCGTGTCATCATCCCCGGATGCATCGGCGGGCGCATGGTCAAGTGGCTCAAGCGCATCATCGTCACACCCGCGGAGTCGGACAACT
ACTACCATTACAAGGACAACCGGGTCTTGCCGTCCCATGTTGACGCCGAGCTCGCCAACGCAGAAGCGTGGTGGTACAAGCCGGAGTA
CATGATCAACGAGCTCAACATCAACTCGGTGATCACCACGCCGGGCCACGACGAGATCCTGCCCATCAACGCCGTCACCACGCAGCGC
GGCTACACCATGAAAGGATACGCCTACTCCGGCGGTGGCAAGAAGGTGACAAGGGTGGAGGTGACGCTCGACGGCGGCGAGACATG
GCTGGTGTGCGCGCTCGACCACCCGGAGAAGCCGAACAAGTACGGCAAGTACTGGTGCTGGTGCTTCTGGTCCGTCGAGGTCGAGGT
CCTGGACCTCCTGGGCGCCAAGGAGATCGCCGTCCGCGCCTGGGACCAGTCGCTCAACACCCAGCCCGAGAAGCTCATCTGGAACCTC
ATGGGTATGATGAACAACTGCTGGTTCAAGGTGAAGGTGAACGTGTGCCGTCCGCACAAGGGCGAGATCGGTCTGGTGTTCGAGCAC
CCGACGCAACCGGGCAACCAGGCCGGCGGATGGATGGCGCGGCAGAAGCACATGGAGACGGCGGAGGCGGCGGCGCCGGGTCTCA
AGCGCAGCACGTCCACGCCCTTCCTCAACACGGCCGGGGACGCCAAGCAGTTCACCATGTCGGAGGTGCGCAAGCACGCGTCGCAGG
AGTCGGCGTGGATCGTCGTGCACGGCCACGTCTACGACTGCACCAAGTTCCTCAAGGACCACCCGGGCGGCGCCGACAGCATCCTCAT
CAACGCCGGCACCGACTGCACGGAGGAGTTCGACGCCATCCACAGCGACAAGGCCAAGGCGCTGCTCGACACGTACCGCATCGGGGA
GCTCATCACCACGGGCACCGGCTACACCTCCGACAACTCCGTGCACGGCGGGTCCGTGCTCTCCCACCTGGCCCCCATCCGGGAGGCCA
TCCGTGCACCGGCGCCCGTGGCGCTCTCCAACCCGCGCGACAAGATCCGGTGCCGCCTCGTCGACAAGAAGACCCTGTCACGCGACGT
GCGCCTGTTCCGCTTCGCTCTGCCGTCGGCGGACCAGGTGCTCGGCCTCCCCATCGGGAAGCACATCTTCGTGTGCGCCGCCATCGACG
GGAAGCTCTGCATGCGCGCGTACACGCCGACGAGCATGGTGGACGAGATCGGCCACTTCGACCTGCTCGTCAAGGTCTACTTCAAGAA
CGAGCACCCGAAATTCCCCAACGGCGGGCTCATGACGCAGTACCTGGAGTCGCTGCCCGTCGGGTCCTACATCGACGTGAAGGGCCCC
CTCGGCCACGTCGAGTACACCGGCCGCGGCAACTTCGTCATCAACGGCAAGCAGCGCCACGCGCGCCGCCTCGCCATGATCGCCGGGG
GGAGCGGGATCACGCCCATGTACCAGGTCATCCAGGCCGTGCTGCGCGACCAGCCGGAGGACACCACGGAGATGCACCTCGTGTACG
CCAACCGGACGGAGGACGACATCCTCCTCCGCGACGAGCTGGACCGGTGGGCCAAGGAGTACCCGGACAGGCTCAAGGTCTGGTACG
TGATCGACCAGGTGAAGCGGCCCGAGGACGGGTGGAAGTACAGCGTCGGCTTCGTCACGGAGGCCATCCTCAGGGAGAACGTCCCCG
AGGGCGGAGACGACACGCTGGCACTCGCATGCGGGCCGCCGCCCATGATCCAGTTCGCCATCAAGCCCAACCTGGAGAAGCTCAAGT
ACGACTTGGAAAGTTCCTTCATCGCCTTCTAAGTTCTAAGTAAGAACCATTAATTACCACGCACTTTCAGATTTGTATGTATATAGGTGTG
TGTCCAAAACCAGTCGAATTGCCTATGCCATCGTTACATGAGATGGGTATTATATATACCGGAGGAAGGTTTGTAAGGTGTACAATTGT
ACTCTTTGCTCTAGTCTTAGAGACTAGAACACGTACATAGGGGGTCATATTTTGCATGCCCTCAGAATGTCAAGTTCTGACTTCTGAGGA
GGCTGCTTCTTTGTCCTGTTACTGTATACTGCTAGCAGGTAACCATTGTTGTTACCTTGGGACCGGGATGTCAGAATATGTAAGCTTAAT
TGTTTGAATGAATGAAGAA

(e) TRINITY_DN20338_c0_g2 Trans_ID=TRINITY_DN20338_c0_g2_i1 2060bp (***Os01g0357100***)

CAACCACACGCTGGACACCCATGGCCACCGTCACAGCACAGCAGCAGCAGAACCGTAGCTACCACAGCCAGCAGCCATGGCCTCCTCC
GCATCCCTGCAGCGTTTCCTCCCGCCCTCCCCGCACGCGGCGGCGTCGTCTCGCCGGCGCCCCGGCCGCGCCCGCCCCGTGCAGTGCCA
GGCCGTCACCACCTCGTCGTCGCCCTCGCCGCCGGCCGTCTCGGAGGAGCGGCTGGAGCGGAGGGTGGAGCAGCGGGAGGGCGGGT
ACTGGGTGCTCAAGGAGAAGTTCCGGACGGGGCTGAACCCGCAGGAGAAGGTGAAGCTGGAGAAGGAGCCCATGGGGCTGTTCATG
GAGGACGGCATCAAGGAGCTCGCCAAGGTGCCCATGGAGGAGATCGACGCCAACAAGCTCTCCAAGGACGACGTCGACGTCCGCCTC
AAGTGGCTCGGGCTCTTTCACCGCCGCAAGCACCAGTACGGCCGCTTCATGATGCGTCTGAAGCTGCCGAACGGCGTGACGACGAGCG
AGCAGACGCGGTACCTTGCGAGCGTGATCGACGCGTACGGCGAGAACGGGTGCGCCGACGTGACCACCCGGCAGAACTGGCAGATCC
GCGGCGTCACGCTCCCCGACGTGCCGGCCATCCTCGATGGGCTCCGCGCCGTCGGGCTCACCAGCCTGCAGAGCGGCATGGACAACGT
GCGCAACCCGGTGGGCAACCCGCTCGCCGGCATCGACCCCGACGAGATCGTCGACACGCGCCCTTACACCAACCTGCTCTCCTCCTACA
TCACCAACAACTCACAGGGGAACCCGGAGTTCACCAACCTGCCGAGGAAATGGAACGTCTGTGTGGTGGGCACGCACGACCTGTACG
AGCACCCGCACATCAACGACCTGGCCTACATGCCGGCCGTCAAGGACGGCAGGTTCGGGTTCAACCTGCTGGTGGGCGGGTTCATCAG
CCCCAAGAGGTGGGGCGAGGCTCTGCCGCTCGACGCCTGGGTCCCCGGCGACGACATCCTCCCCGTGTGCGGGGCCATTCTCGAGGC
GTTCAGGGACCTCGGCAACAGGGGCAACCGCCAGAAGACGCGCATGATGTGGCTCATCGATGAACTCGGGATGGAAGTGTTCCGGTC
GGAGGTGGAGAAGCGGATGCCGAACGGGGTGCTGGACCGCGCCGCGCCGGAGGACCTGATCGACAACACCTGGCAGCGGCGCGACT
ACCTGGGCGTGCACCCGCAGAAGCAGGAAGGCCTGTCGTACGTGGGCCTGCACGTGCCCGTGGGCCGTGTGCAGGCCTCCGACATGT
TCGACCTCGCGCGCCTCGCCGACGAGTACGGCTCCGGCGAGCTCCGGCTCACGGTGGAGCAGAACATCGTGCTCCCCAACGTGAAGAA
CGAGCGGCTCGACGCGCTGCTCGCGGAGCCGCTGCTGCAGAAGCTCTCCCCGCAGCCGTCGCTGCTGCTCAAGGGCCTCGTGGCGTGC
ACGGGCAACCAGTTCTGCGGCCAGGCCATCATCGAGACCAAGATGCGCGCGCTCAACGTGACGCAGGAGGTGGAGAAGCGCGTCTCG
GTGCCACGGCCCGTGCGCATGCACTGGACCGGGTGCCCCAACAGCTGCGGCCAGGTGCAGGTCGCCGACATCGGCTTCATGGGGTGC
CTCACCAAGGACAGCAACGGCAAGATCGTCGAGGCGGCGGATATCTTCGTCGGCGGCCGCGTCGGCAGCGACTCGCATCTCGCGGAC
GTGTACAAGAAGTCCGTGCCGTGCGACGAGCTTGCGCCCATCGTGGCTGACCTCCTCGTTGAGAAATTTGGGGCCGTGCCGAGGGAGA
GGGAGGAGGATGAGGAGTAGGAACACCTACTGCGGCTGTTCCGCTGATCTTCGTTCCTTGACGTACTCCGAGTTGAGTTACCGACCGT
TTGTTGTAAAGTAGATGATGCCCTCTTCCCTGGCACGGCTAAATGTTCCACGATTGTTTTTGATGTGTAGTCCACAGTTTTCCAGTTCACA
CGTTACAGTGAATGCTGATAAATAAAGTAACACGCTGCAATACAGG

(f) TRINITY_DN22941_c0_g1 Trans_ID=TRINITY_DN22941_c0_g1_i5 932bp (***CA1***)

GTTCGAGGACTTCAAGGTCAATGTATACAACAAGAAGCCGGAGCTGTTCGAGCCGCTCAAGGAGGGCCAGGCACCTACGTACATGGT
GTTTGGCTGCGCCGACTCCCGATGCTGCCCTTCAGTGACCCTCGGCCTGCAGCCGGGCGAGGCCTTCACCATGCGCAACATTGCCAGC
ATGGTCGGTCCCTATGACAAGAACAAGTATACTGGCACCGGGTCCGCCATCGAGTACGCCGTCTGCGCTCTCAAGGTCTCAGTCATTG
TGGTCATTGGCCACAGCCGCTGCGGTGGTATCAAGGCCCTCCTCTCCATGGAGGAAGGTGCACCCGACAACTTCCACTTCGTTGAGGA
CTGGGTCAGGATCGGCTTACCCGCCAAGAAGAAGGTGCAGACTGAGTGCAAGGCATTGCCGTTCGATGCACAGTGCAGTGTCTTGGA
AAAGGAGGCCGTCAATGTGTCCCTTGACAACCTAAAGACTTACCCATTCGTCAAAGAAGGAGTGGAAAATGGGAGCCTCAAGCTGGT
CGGCGCCCACTACGACTTCGTCAACGGCAAGTTCGAGACATGGGACGCTCCAACTCCTCCTCCTTCCAAGGCTGTGGATGCTGTTGCA
CGCTTGAAGACTGGGTTCCAAAACTTCAAGATCGATGTCTACGACAAGAAGCCAGAATTATTCGAGCCACTTAAGGAGGGCCAGGCA
CCCACGTTCATGGTGTTCGCTTGTGCGGACTCCCGGTGCTGCCCTTCGGTAACCCTCCGCCTGCAACCCGGTGAGGCCTTCACCATACG
CAACATTGCCAGCATGGTCGGACCCTACGACAAGACCAAGTACACCGGCATTGGGTCTGCCATCGAGTATGCCGTCTGCGCGCTCAAG
GTCTCATGCATCGTGGTCATTGGCCACAGCCGTTGTGGTGGTATCAGGGCCCTCC
